# Analysis of factors influencing the organizational capacity of Institutional Review Boards In China: a crisp-set Qualitative Comparative Analysis based on 107 cases

**DOI:** 10.1186/s12910-023-00956-3

**Published:** 2023-09-26

**Authors:** Lu Lu, Shuwen Shi, Bojing Liu, Chanjuan Liu

**Affiliations:** 1 College of Education, Zhejiang Normal University, JinHua, 321000 Zhejiang People’s Republic of China; 2 Institute of Medical Humanities, Wenzhou Medical University, Wenzhou, 325000 Zhejiang People’s Republic of China; 3 Outpatient Department of Zhejiang Eye Hospital, HangZhou, 310000 Zhejiang People’s Republic of China

**Keywords:** Institutional Review Boards, Organizational capability, Qualitative Comparative Analysis, Influencing factors

## Abstract

**Background:**

Institutional Review Boards (IRBs) play a vital role in safeguarding the rights and interests of both research participants and researchers. However, China initiated the establishment of its own IRB system relatively late in comparison to international standards. Despite commendable progress, there is a pressing need to strengthen the organizational capacity building of Chinese IRBs. Hence, this study aims to analyze the key factors driving the enhancement of organizational capacity within these committees.

**Method:**

The cross-sectional survey for this research was conducted from July 2020 to January 2022. Following the statistical grouping based on the "2020 China Health Statistical Yearbook", a systematic investigation of IRBs in various provinces of China was carried out. In-depth interviews and questionnaire surveys were conducted with the chairpersons and administrative executives (or secretaries) of highly cooperative IRBs. Subsequently, data were collected from 107 IRBs. Qualitative Comparative Analysis (QCA) was employed as the method to analyze the factors influencing the organizational capacity of medical ethics committees and explore the diverse combinations of these factors.

**Results:**

Through a singular necessary condition analysis, the variable "protection of rights and interests" emerges as a critical factor contributing to the robust construction of Institutional Review Boards Institutional Review Boards (IRBs). Conversely, the variables of "lack of member ability, absence of review process, and deficiency in the supervision mechanism" collectively constitute a sufficient condition leading to weaker IRB construction. The state analysis uncovers three interpretation modes: member ability-oriented (M1), system process-oriented mode (M2), and resource system-oriented mode (M3).

**Conclusions:**

The results of this study are effectively explicable using the "Triangular Force" model proposed for the hypothesis of IRBs' organizational capacity, which provides a solid foundation for the development of organizational capabilities in IRBs. To enhance the organizational capacity of IRBs in China, it is imperative to elevate the competence of committee members and strengthen team development. This can be achieved by establishing a comprehensive regulatory framework and refining procedural protocols. Moreover, clarifying the organizational structure and optimizing resource allocation are essential steps in bolstering the overall organizational capabilities of these committees.

## Background

Presently, in China, the accepted and widely implemented classifications encompass Hospital Ethics Committees (HECs), Institutional Review Boards (IRBs), and Medical Ethics Committees (MECs) established by national government bodies and medical organizations. In specific applications, MECs are established within national administrative departments and primarily serve as ethics consultation bodies for significant ethical concerns, such as the "National Health and Health Commission Medical Ethics Expert Committee." However, confusion and discussion often arise concerning the two types of medical ethics committees, namely, HECs and IRBs. It can be discerned that HECs belong to the hospital's consultation mechanism and are responsible for providing ethical consultation on obligatory ethical choices encountered in clinical practice within the hospital. Conversely, IRBs refer to the ethical review of clinical trials and medical research projects involving human subjects. IRBs possess the authority to review and approve these projects, and their approval is imperative for the projects to proceed [1]. The subject matter explored in this article pertains to the entity entrusted with the responsibility of conducting ethical reviews for clinical trials and medical research projects involving human subjects, commonly referred to as Institutional Review Boards (IRBs). In China, Grade III hospitals are required to set up IRBs, so most mature IRBs in China are affiliated with tertiary hospitals, but they are independent.

In recent years, the rapid advancement of scientific technologies, such as gene editing, artificial intelligence, and organ transplantation, has yielded significant benefits for humanity. However, it has also introduced novel risks and uncertainties, thereby presenting unprecedented ethical challenges [2]. To effectively address these challenges, the establishment of IRBs is imperative to ensure the implementation and enforcement of relevant laws, regulations, and ethical standards [3]. Consequently, there is an urgent need to enhance and strengthen the organizational capacity of IRBs. Enhancing the capabilities of IRBs has emerged as a pivotal area of current research. In comparison to international institutional review boards, China embarked on this endeavor relatively later. As the concept of Ethics Committees was introduced to China, its understanding has evolved over time. Initially, there were policy consultations concerning the establishment of ethical committees in the medical field. Subsequently, discussions revolved around their formation and establishment processes and have now shifted toward strategies for further enhancing their construction [4].

However, in recent years, significant achievements have been made by Chinese IRBs in terms of protecting rights and interests, system development, and procedural establishment. These accomplishments can be attributed to the efforts of Chinese government departments, academic organizations, and experts at various levels. Nonetheless, the organizational capacity of Chinese IRBs remains relatively weak. Previous research has predominantly focused on individual factors, such as the establishment of IRB systems and the development of procedural frameworks. Moreover, strategies proposed for IRB construction have often relied on case analysis or theoretical normative analysis, lacking systematic comparisons and comprehensive studies across cases [5]. Considering the perspective of organizational capacity, the establishment of IRBs in China is influenced by multiple factors from different levels and stakeholders, resulting in complex causal relationships. Therefore, this study adopts a Qualitative Comparative Analysis(QCA)" approach to examine this issue. It aims to analyze the current operational status of institutional review boards in China from an organizational capacity perspective, explore the factors and their combinations that influence IRB organizational capacity, identify the driving factors for strengthening IRB organizational capacity in China, and critically analyze the current management situation of IRBs in the country. The ultimate goal is to enhance the comprehensive capabilities of Chinese IRBs, facilitate their effective functioning, and advance ethical review work in China.

## Methods

### The reason for selecting Qualitative Comparative Analysis (QCA)

First, QCA differs from single-case analysis because it is a cross-case research method. The general criteria for case selection require the case population to exhibit sufficient homogeneity (i.e., cases must possess adequate background or characteristics) and maximum heterogeneity within the case population (i.e., a significant degree of diversity among cases). In this study, IRBS in domestic Grade IIIA hospitals were selected as the research object, and these hospitals all operated within the framework of relevant health management policies in China. These cases are representative and meet the requirements of sufficient homogeneity. Furthermore, due to the differing circumstances of IRBs across various Chinese hospitals, such as variations in their establishment time, personnel numbers, and the independence of research subjects, the QCA method's prerequisite for maximum heterogeneity of research objects is met. Second, unlike quantitative analysis, QCA usually requires 10 to 80 cases, which makes QCA more advantageous when analyzing small samples [6, 7]. This study was of the ethics committees of the Grade IIIA hospitals in China, and the number was limited; therefore, the study sample was a small sample, which met the requirements of the method.

The third reason is that the capacity building of a medical ethics committee is influenced by various factors, including both personal and organizational environmental factors. This study specifically focused on the organizational environmental factors within the institution where the IRB is situated, such as the availability of resources, organizational culture, and educational background. In addition to the complexity of personal factors, if regression analysis focusing on the measurement of net effects is used, it is easy to cause multicollinearity and the consumption of degrees of freedom. Multiple linear regression was used; however, QCA breaks through this net effect and combines various conditional factors. We used a combination to explain the result with the greatest strength.

### Sample selection and data collection

To ensure the reliability and validity of the measurement instrument, this study utilized a well-established questionnaire previously developed by esteemed Chinese scholars [8–10]. Subsequently, the questionnaire underwent modifications based on expert recommendations. Prior to the main data collection, a preliminary investigation was carried out to administer the questionnaire. A presurvey was conducted in hospitals located in Zhejiang Province, China, including The First Affiliated Hospital of Wenzhou Medical University, the Eye Optometry Hospital of Wenzhou Medical University, the Second Affiliated Hospital of Wenzhou Medical University, and the First Affiliated Hospital of Zhejiang University. After conducting interviews with members and secretaries of the IRBs and gaining a comprehensive understanding of the internal structure of the aforementioned IRBs, certain questions and options in the questionnaire were modified to develop the survey instrument used in this investigation.

The research team conducted the study from July 2020 to January 2022. The distribution of public comprehensive tertiary Grade III A hospitals in various provinces of China, as reported in the "2020 China Health Statistical Yearbook," was used as a basis for selecting the participating institutions. It is a requirement in China that IRBs be affiliated with tertiary hospitals. To collect data, structured questionnaires were distributed to 550 IRB chairpersons and administrative executives (or secretaries) from these institutions through on-site visits, phone interviews, and video conferences. The team received 110 valid questionnaires in response. Additionally, interviews were conducted with highly cooperative IRBs during the survey period to complement and verify the data obtained from questionnaires. Second-hand data were also utilized to further supplement and validate the interview and questionnaire data. This process ensured the accuracy and reliability of the information. During the data verification process, the team used second-hand data to clarify or supplement individual values in the questionnaire with partial data ambiguities or missing values. As a result, three IRBs with missing data were excluded from the study, leading to an effective sample size of 107 and an effective questionnaire response rate of 97.3%.

### Determining the variables: literature analysis

We used "Medical Ethics Committee", “Ethics Committee”, or "Institutional Ethics Committee" as the subject headings, searched according to topics or keywords in the CNKI (China national knowledge infrastructure) database, and selected the core journals and CSSCI (Chinese Social Science Citation Information) journals as the search source. A total of 20 documents were searched. Considering that China promulgated the "Measures for the Ethical Review of Biomedical Research Involving People" in December 2016, and it was officially put into operation, we set the time node for the selection of the literature from 2017 to the present. In the obtained documents, after removing duplicates and redundant and not highly relevant documents, we found that many documents put forward external suggestions for establishing regional ethics committees, strengthening external evaluation and certification, etc.

The focus of this article was on committee organization. The internal factors affected the capacity, and thus, only the variables mentioned in 11 documents concerning the construction of the Institutional Review Board were analyzed. At the same time, we fully studied and considered the requirements of China’s relevant laws and regulations on ethics review. Under this premise, we analyzed and contrasted the forward-looking and superior evaluation content in the international evaluation standards (see Table 1), which affect the operation of the medical ethics committee. The internal influencing factors were refined and divided into six categories (see Table 2).

**Table 1:** The regulations and international guidelines on ethical review. Files from the official website of the Chinese government and the US Office for Human Research Protections and World Medical Association

	Name	Issuer	Time
China	“Guidelines for the construction of clinical research ethics committees involving human subjects”	Office of Medical Ethics Expert Committee of China National Health Commission	in 2019
	“Measures for Ethical Review of Biomedical Research Involving Human Subjects”	China National Health and Family Planning Commission	in 2016
	“Drug Administration Law of the Peoples Republic of China”	The Twelfth Meeting of the Standing Committee of the 13th National Peoples Congress of China	in 2019
	“Guiding Principles for Ethical Review of Drug Clinical Trials”	China State Food and Drug Administration	in 2015
	“Administrative Measures for the Clinical Application of Medical Technology”	National Health Commission	in 2018
	“Administrative Measures for Drug Registration”	China State Administration for Market Regulation	in 2007
	“Measures for Ethical Review of Biomedical Research Involving Human Subjects (Trial)”	National Health and Family Planning Commission	in 2016
	“Guiding Principles for Clinical Trials of In Vitro Diagnostic Reagents”	State Food and Drug Administration	in 2014
	“Regulations on Clinical Trials of Medical Devices”	State Food and Drug Administration	in 2004
	“Pharmaceutical Clinical Trial Quality Management Standards”	State Food and Drug Administration	in 2003
	“Regulations for the Implementation of the Drug Administration Law of the Peoples Republic of China”	Order of the State Council of the Peoples Republic of China	in 2016
International	The Nuremberg Code	Nuremberg Military Court	in 1949
	The Belmont Report	Belmont Convention Center	in 1964
	The Declaration of Helsinki	World Medical Association	in 2013
	“International Ethical Guidelines for Human Biomedical Research” (CIOMS)	International Committee of Medical Scientific Organizations	in 2002

**Table 2:** Analysis of factors affecting Institutional Review Board. References from China National Knowledge Internet (CNKI)

Classification	Author and Time
Related to monitoring mechanism	(Xue, D., 2019) [11], (Li et al., 2017) [12], (Zhang et al., 2017) [13], (Wen et al., 2018) [14], (Wang, J. and Xin, B. 2019) [15], (Zhou et al., 2017) [16]
Related to member ability	(Wen et al., 2018) [14], (Fan et al., 2017) [17], (Wang, J. and Xin, B., 2019) [15], (Zhang et al., 2017) [13], (Zhou et al., 2017) [16]
Related to office resources	(Yu et al., 2020) [18], (Zhang et al., 2017) [13]
Related to rights protection	(Li et al., 2017) [12], (Jiang et al., 2017) [19]
Related to the review process	(Wang, H. B. and Wang, Y. R., 2017) [20], (Jiang et al., 2017) [19], (Fan et al.,2017) [17], (Yu et al., 2020) [18], (Wang, J. and Xin, B. 2019) [15], (Zhang et al., 2017) [13]
Related to rules and regulations	(Wen et al., 2018) [14], (Zhou et al., 2017) [16], (Zhang et al., 2017) [13]

This study refers to the relevant Chinese foreign policies and regulations of the medical ethics committee and relevant authoritative documents, combined with the actual situation of the on-site interview data and case responses, and summarizes the relevant variables that affect the capacity building of the medical ethics committee in the member structure, office resources, and systems with six factors, including the review process, internal and external supervision, and rights protection. The capacity building of the Institutional Review Board is the result of the combined effect of many factors, which is an ongoing problem.

However, how the membership structure, office resources, rules and regulations, review process, rights protection, supervision mechanisms, and other factors together affect the capacity building of the Institutional Review Board is an open question. Therefore, this article attempts to explore the joint effects of the above variables on the capacity building of IRBs and to explain the possible connections between different variables.

### Variable measurement and calibration

The condition variables of this study encompass relevant organizational factors that impact the functioning of the medical ethics committee, evaluated based on their compliance with industry regulations during the committee's operation. The outcome variable pertains to the effectiveness of the ethics committee's construction, which is a categorical variable. Considering the distinctive characteristics and applicable requirements of various qualitative comparative analysis methods, as well as the data attributes and research objectives, the crisp-set Qualitative Comparative Analysis (csQCA) method was employed for cross-case analysis of the system. The samples were subjected to clear-set bifurcation, followed by iterative calibration and testing [21]. We conducted a systematic data collection and literature search to comprehend the regulations and international guidelines that the institution must adhere to concerning ethics review. Additionally, we examined the existing relevant content pertaining to the management and establishment of ethics committees. In this study, various evaluation criteria were integrated, and we referenced the relevant provisions in the "Measures for the Ethical Review of Biomedical Research Involving People," which was promulgated by the Chinese government in December 2016. To ensure clarity and precision, we followed a qualitative comparative analysis approach utilizing the binary method [22]. Values were assigned to the selected cases based on their adherence to established norms.

After repeated calibration and testing, 29 cases with good medical ethics committee construction were obtained, accounting for 27.1% of the total number of cases, and 78 cases with poor construction were obtained, accounting for 72.9% of the total number of cases. When assigning the result variable, this article counted each case that met a condition variable as “1 point” and added “1 point” to those that passed the international assessment, for a total of two points [23] (see Table 3).

**Table 3:** Condition variable assignment

Condition Variable	Measurement Condition	Classification	Coding
Member ability	Reasonable structure, independent members, independent consultants, etc., meet the requirements	All meet	1
		Does not meet	0
Office resources	Office conditions, meeting rooms needed for review meetings; dedicated medical ethics committee management room; organizations provide necessary financial funds, etc	All meet	1
		Does not meet	0
Rules and regulations	Does the charter, work system, and standard review process exist?	All meet	1
		Does not meet	0
Review process	The review methods and review records are all compliant and complete	All meet	1
		Does not meet	0
Monitoring mechanism	Internal: there is supervision within the organization, etc	All meet	1
	External: information disclosure, acceptance of industry supervision, etc	Does not meet	0
Rights protection	Protection of rights and interests of subjects; protection of rights and interests of experimenters	All meet	1
		Does not meet	0
Outcome variable	Compliant with the six variables will be counted as 1 point, and 1 point will be added to those who pass domestic and foreign verification	0–3	0
		4–7	1

## Results

### Necessity analysis of individual conditions

The QCA analysis followed a four-step approach supported by the software fsQCA. Consistent with mainstream QCA research, this article first examined whether a single condition (including its noncollection) constitutes a necessary condition for a complete merger. From the perspective of set theory, the necessity analysis of a single condition is to test whether the result set is a subset of a certain condition set. If condition X (a single condition or a combination of conditions) is a sufficient condition for Y, then the fuzzy set score of X should be less than or equal to the fuzzy set score of Y, and the consistency index should be greater than 0.8. When the consistency level is greater than 0.9, the condition can be considered a necessary condition for the result [24–26].

Table 4 shows the test results of the necessary conditions for the establishment of a better Institutional Review Board using fsQCA3.0 software analysis. The consistency of the rights protection exceeded 0.9. It can be seen that rights protection is a necessary condition for the results (the consistency is 0.93), followed by the review process and office resources (consistency is 0.72). In view of the causal asymmetry of the QCA method, Table 5 shows the results of the analysis of the necessary conditions for the poor construction of an Institutional Review Board. The lack of member ability, the lack of review process, and the lack of supervision mechanisms are sufficient conditions for poor IRBs. The consistency of rights protection was 0.78, indicating that the construction of the Institutional Review Board is relatively good and poor, and the Institutional Review Board in terms of rights protection is relatively standardized.

**Table 4:** Necessity test for better construction of medical ethics committee

Condition Variable	Consistency	Coverage
Member ability	0.620690	0.666667
~ Member ability	0.379310	0.137500
Office resources	**0.724138**	**0.320000**
~ Office resources	0.275862	0.256098
Rules and regulations	0.724138	0.396226
~ Rules and regulations	0.275862	0.148148
Review process	**0.724138**	0.636364
~ Review process	0.275862	0.108108
Monitoring mechanism	0.620690	0.545455
~ Monitoring mechanism	0.379310	0.148649
Rights protection	**0.931035**	0.306818
~ Rights protection	0. 068966	0.105263

“ ~ ” means that a factor does not appear or is "not"

**Table 5:** Necessity test of poor construction of medical ethics committee

Condition Variable	Consistency	Coverage
Member ability	0.115385	0.333333
~ Member ability	**0.884615**	**0.862500**
Office resources	0.217949	0.680000
~ Office resources	0.782051	0.743902
Rules and regulations	0.410256	0.603774
~ Rules and regulations	0.589744	0.851852
Review process	0.153846	0.363636
~ Review process	**0.846154**	**0.891892**
Monitoring mechanism	0.192308	0.454545
~ Monitoring mechanism	**0.807692**	**0.851351**
Rights protection	**0.782051**	**0.693182**
~ Rights protection	0.217949	0.894737

“ ~ ” means that a factor does not appear or is "not"

### Adequacy analysis of conditional configuration

Different from the abovementioned necessary condition analysis, the configuration analysis attempts to reveal the sufficiency analysis of the results caused by different configurations composed of multiple conditions. From the perspective of set theory, we explored whether the set represented by the configuration composed of multiple conditions is a subset of the result set. Conditional combination analysis uses a combination method to analyze the influence of different combinations on the outcome variable when a single variable does not meet the necessary conditions. Based on existing research, this paper set the consistency threshold to 0.8 and the case frequency threshold to 1 and calculated the complex solution, simple solution, and intermediate solution of the location selection result. We combined the intermediate solution and a simple solution to explain the obtained conditional configuration (see Table 6).

**Table 6:** A good intermediate solution for medical ethics committee construction

Path	Condition Combination	Original Coverage	Unique Coverage	Consistency
P1	Member ability*rules and regulations*review process*rights protection	0.344828	0.137931	1
P2	Office resources*rules and regulations*review process* ~ supervision mechanism*rights protection	0.103448	0.0689656	1
P3	Member ability* ~ office resources*rules and regulations*supervision mechanism*rights protection	0.206897	0.0344828	1
**P4**	Member ability* ~ office resources*review process*supervision mechanism*rights protection	**0.275862**	**0.103448**	**1**
**P5**	~ Office resources*rules and regulations*review process*supervision mechanism*rights protection	**0.310345**	**0.137931**	**1**
P6	~ Member ability*office resources* ~ rules and regulations* ~ review process*supervision mechanism* ~ rights protection	0.0344828	0.0344828	1
P7	Member ability*office resources* ~ rules and regulations* ~ review process*supervision mechanism*rights protection	0.0689655	0.0689656	1

Solution coverage: 0.793103 solution consistency: 1*.*“ ~ ” means logical "not", "*" means logical "and"

The original coverage rate indicates the proportion of cases that can be explained by the condition combination in the total cases; the net coverage rate indicates the proportion of cases that can only be explained by the condition combination in the total cases; and the consistency indicates the path (condition or condition combination) reliability or suitability [7].

By operating the truth table, the coverage of the solution is 1, and the conditional combination can explain all cases (see Table 5). There are seven paths to promote the "good construction of IRBs".

Among the seven combined paths, the unique coverage of P1, P4, and P5 is approximately 0.13, 0.10, and 0.13, respectively. The unique coverage of P2 and P7 also reached approximately 0.068. These four paths can explain approximately 49.6% of cases and have strong explanatory power for the causal mechanism of Institutional Review Board. To improve the explanatory power of the final path combination, based on the basic principles of Boolean operations, this study transformed Table 6 into Table 7 with a more concise condition similarity in the path combination and modeled and summarized the seven well-established paths of the medical ethics committee.

**Table 7:** Good condition configuration for medical ethics committee construction

	**Resource System-Oriented Model (M3) Institutional**			**Member Capability-Oriented Model (M1)**		**Process-Oriented Model (M2)**	
	**P6**	**P7**	**P1**	**P3**	**P4**	**P5**	**P2**
Monitoring mechanism	●	●	–	●	●	●	×
Office resources	●	●	–	×	×	×	●
Member ability	×	●	●	●	●	–	–
Rules and regulations	×	×	●	●	–	●	●
Review process	×	×	●	–	●	●	●
Right Protection	×	•	•	•	•	•	•
Unique coverage	0.1034484			0.2758618		0.2068966	

“•” means "general conditions", “●” means "core conditions", “ × ” means "conditions do not appear", and "–" means "corresponding conditions with paths do not matter". Simple solution and intermediate solution. The condition that appears at the same time is the "core condition", and the condition that only appears in the intermediate solution is the "general condition", using the format of Fiss (2011)

We refined the shared conditions or combination of conditions and built a more explanatory model. In the end, three modes are summarized: the member ability-oriented (M1), institutional process-oriented mode (M2), and resource system-oriented mode (M3). The combination of these three models can explain all cases, and the unique coverage reached approximately 0.59 in total. Each model has strong explanatory power, as follows:

1. The first interpretation model is the member ability model (M1), which includes path 1, path 3, and path 4. The basic expression after Boolean simplification is M1 + member ability*rights protection*[rules and regulations*review process +  ~ office resources*supervision mechanism*(rules and regulations + *review process)]. M1 covers 24 cases. In the sample case, the basic condition (combination) is the members’ ability and rights protection. That is, under the premise of the protection of member capabilities and rights and interests, there is the existence of rules, regulations and review processes or a lack of office resources, but the existence of a supervisory mechanism, the coexistence of rules, regulations and review processes, and the capacity building of the Institutional Review Board are also good.
2. The second interpretation model is the system process-oriented model (M2), which includes Path 5 and Path 2. The basic expression after Boolean simplification is M2 = rules and regulations*review process** rights protection (office resources* ~ supervision mechanism +  ~ office resources*supervision mechanism). M2 covers 12 sample cases, and the basic conditions (combination) are the review process, rules and systems, and rights protection. That is, regardless of the ability of the members, only one type of office resource and supervision mechanism are qualified. When there are rules and regulations, review processes, and rights protection, the capacity building of the medical ethics committee is also relatively good.
3. The third interpretation model is the resource system-oriented model (M3), which includes path 6 and path 7. The basic expression after Boolean simplification is M3 = office resources*supervision mechanism* ~ rules and regulations * ~ Review process (~ member ability ~ rights protection + member ability*rights protection). M3 covers three cases. The basic condition (combination) is office resources, lack of supervision mechanism, rules and regulations, and lack of review process. That is, when the rules and regulations and review process are missing, regardless of the ability of members and the protection of rights and interests, the existence of office resources, and supervision mechanisms, the capacity building of the medical ethics committee is also better.

### Robustness test

This article references the Fiss and Zhang Mings method to increase the consistency level from 0.8 to 0.85 for robustness testing [27, 28]. After adjusting the consistency level threshold from 0.8 to 0.85 in fsQCA, the case frequency was still 1, and the overall solution was consistent after analysis. The level of performance was 1, which still has a good explanation strength. The coverage of the overall solution is the same as before. The configuration after adjusting the consistency threshold is consistent with the configuration before the adjustment. Therefore, after increasing the adjustment consistency threshold, the result is still robust.

## Discussion

### Model construction

**Fig. 1:**
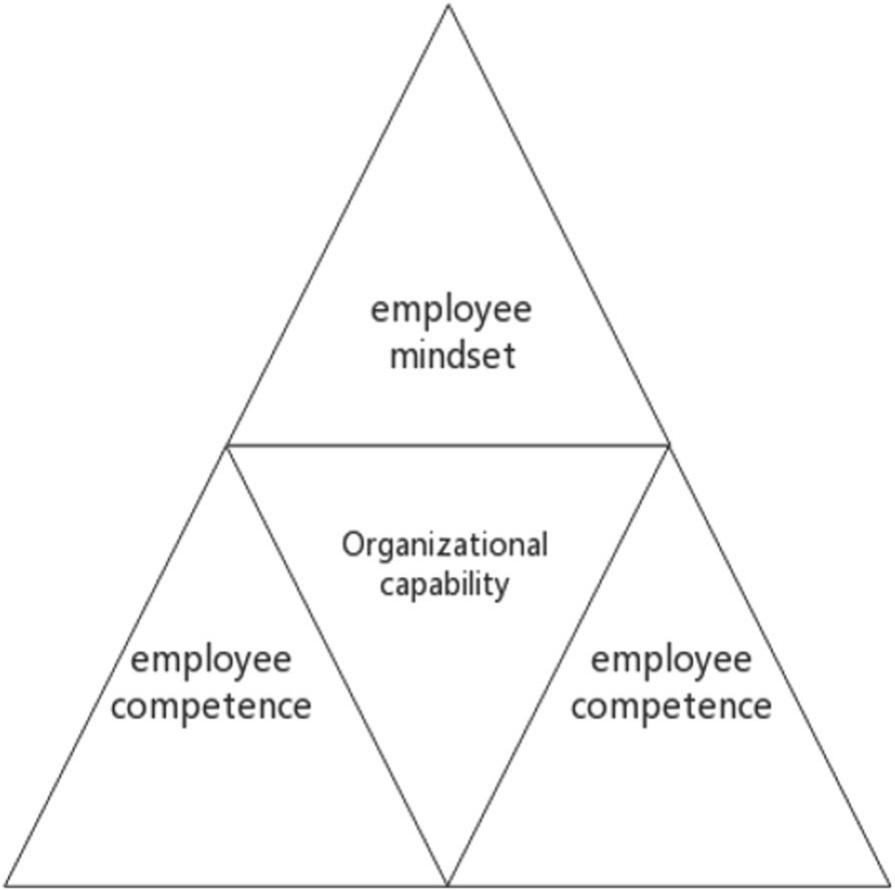
Triangular framework of organizational capabilities

**Fig. 2:**
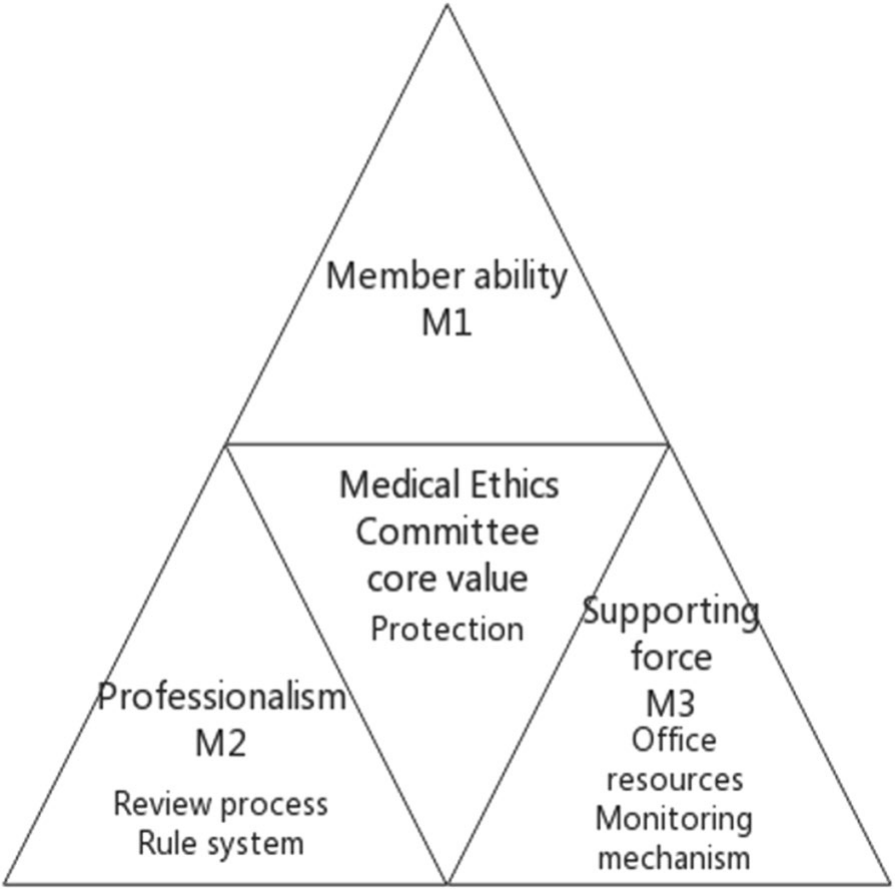
The "Three Forces Model" of IRBs

In this article, we regard the medical ethics committee as an organization and refer to the "triangular framework of organizational capabilities" proposed by Professor Yang Guoan, also known as the "Yang Triangle" model [29]. Guoan proposed that organizational capabilities were inseparable from employee capabilities and employee thinking patterns and indicated that organizational capacity building could not be separated from the three pillars of employee mentality, employee ability, and employee governance (as shown in Fig. 1). Figure 2 shows the "three-force model" for the management of the medical ethics committee as constructed by the author.

The study reveals that the inadequate main factors in IRB management align with the three dimensions of the "Yang Triangle Force" in terms of management. Therefore, the author formulates a hypothetical model of medical ethics committee organizational capabilities, known as the "Three Forces" model, from an organizational perspective. This model represents a reflection and exploration of the Institutional Review Board's management approach, revolving around the core value of the medical ethics committee—the protection of the rights and interests of research subjects. The "Triangle Force" model encompasses three key elements: organizational support (office resources and supervision mechanism), management expertise (review process, rules, and regulations), and the capabilities of medical ethics committee members. The author successfully constructs this "Three Forces Model" to enhance medical ethics committee management.

To enhance or transform organizational capability, simultaneous adjustments should be made in the following three aspects. The construction of an institution's organizational capability relies on the synergistic cooperation of these aspects. A deficiency in any one aspect can lead to the failure of overall efforts. Therefore, for the establishment, construction, or renovation of the IRB, the authors must carefully balance the following three aspects:

### Focusing on the shaping of member ability

Even if committee members are willing to cooperate, they may not have sufficient competence. In this case, organizations should start by providing training and improving their competence. The working ability of the IRB members is the basic ability of IRBs to perform their functions; however, the abilities of the IRB of each organization are not the same. In the actual operation of the organization, the review standards of the ethics committee of each IRB are often different. In the same report, it is not uncommon for an IRB to pass the review without modification but not pass the review in other IRBs. The fundamental reason lies in the ethics of the ethics committee members. There is a gap between quality and ethical review ability. Insufficient ethics knowledge training and education come from different professional hospital ethics committee members with special skills. The necessary knowledge of medical ethics, the ability to understand and execute ethical principles and related laws and regulations, and the degree of understanding and mastery of new medical technologies may be different.

However, as a member of the IRB, the imperfection of ethical knowledge will seriously affect the effective use of the ethical review role of the IRB. The role of the members is not balanced. The members of the committee have different professional backgrounds and understandings of ethical principles and laws [30]. The understanding and grasp of laws and regulations, as well as the comprehension of experimental protocols, vary significantly, leading to substantial differences in the perspective and authority of reviews. IRB members assume the responsibilities of reviewing from diverse angles, which are closely related to their own interests and may even lead to conflicts [31]. It is noteworthy that a considerable portion of IRB members are hospital employees, which could introduce a bias stemming from their affiliation and emotional connections to the organization. Such bias may potentially impact the objectivity and fairness of ethics reviews [32]. Stakeholders, including IRB members, frequently encounter ethical dilemmas arising from potential conflicts between fulfilling their duties and pursuing personal aspirations for professional growth and self-interests. The imperative to advance IRBs is evident in the need to strengthen their skill set, forming a foundational requirement for enhancing their effectiveness and competence. This, in turn, ensures comprehensive support for their pivotal roles and functions.

### Optimize the improvement of the system process

The IRB thinking encompasses the management of professional ethical review work, including the review process, adherence to rigorous procedures, rules, and regulations in accordance with relevant laws and regulations, and prioritizing the protection of rights and interests. The ethical review process should be conducted in a meticulous and orderly manner. Organizational thinking should adhere to standardized practices in executing responsibilities. The establishment of a sound system process can better ensure the independence of ethical organizations and truly reflects the connotation of independence. Independence includes not only the requirements of independent places and supporting facilities but also, more importantly, the rules and regulations of ethical organizations, particularly the standards of ethical organizations. In the implementation of procedures, the institutional process-oriented model (M2) in the abovementioned research and any medical ethics organization must standardize its operation, where the primary task is to continuously improve the construction of a series of rules and regulations with the goal of standardization, institutionalization, and proceduralization. Hospital ethics organizations must strictly abide by various rules and regulations [33]. For ethics organizations and their persons in charge who do not strictly abide by the rules, necessary punishments and the disqualification of evaluation qualifications must be carried out to ensure the standard operation of ethics organizations.

### Attention regarding the construction of resource systems

Even if members have the ability, the organization’s infrastructure, process, and organization’s structure may allow members to lose momentum in manifesting the IRB through a lack of office resources, supervision mechanisms, etc. The prerequisite for the medical ethics committee to have full power in its role is that it has sufficient organizational support—that is, the resource system-oriented model (M3) in the abovementioned research. The IRBs in China are often affiliated with other departments throughout the year, resulting in organizational confusion, insufficient supporting facilities, and a lack of resources. The lack of resources, including facilities, funds, and technology, within the medical ethics committee can be attributed to several factors. First, the institution may have a limited understanding of the IRB, resulting in a lack of scientific planning. Additionally, since the medical ethics committee is not an independent department, obtaining organizational resources becomes challenging [34].

To address this issue, it is crucial to allocate the necessary resources for the committee's work and enhance organizational support. In 2016, China introduced regulations, such as the "Measures for the Ethical Review of Biomedical Research Involving People," which highlighted the need for appropriate remuneration and training for ethics committee members, indicating the requirement for funding from the department or institution that establishes the institutional ethics committee. Proper funding enables the committee to fulfill its responsibilities effectively and ensures that committee members receive compensation and training in accordance with regulations. However, a considerable number of ethics organizations in China still lack the basic funds to maintain their normal operations. In most cases, in hospitals where they are allocated certain funds or project review fees, there are potential risks that affect the independence, objectivity, and fairness of the review results.

It is necessary to strategically plan medical ethics committees and improve the allocation of relevant resources, not only by clarifying the organizational structure of the committees but also by establishing their relative independent management and operational mechanisms. This ensures that the medical ethics committees have their own discourse power and avoids them becoming intermediaries for the transmission of discourse power through "attachment" and affiliation with other departments. Additionally, it is important to define the internal and external entities responsible for regulation, enhance the self-management of ethics committees, and construct a combined internal and external supervision system.

## Conclusions

In conclusion, the "Triangle Force" model represents a profound reflection and exploration of the management approach for medical ethics committees, establishing a synergy that revolves around the core value of protecting the rights and interests of subjects and experimenters. This model offers guidance for optimizing the organizational structure of IRBs and streamlining IRBs management practices. Under the impetus of ethical norms and moral constraints, the effective management and standardization of medical ethics committees can promote the positive development of biomedical technology and scientific advancement in various fields for the benefit of humanity. For future research, it is crucial to continue exploring the influencing factors of IRB and further tailor the improvement of their organizational capacity. Additionally, as IRBs access more foreign resources, official statistical data, and other publicly available information, combined with ongoing research in the related field, the author will strive to address the remaining issues and enhance the scientific rigor and effectiveness of this research area.

## Data Availability

The data that support this study are available from the corresponding author (CV) on reasonable request, subject to privacy and confidentiality commitments.

## Abbreviations

IRBs: Institutional Review Boards
csQCA: Crisp-set Qualitative Comparative Analysis
QCA: Qualitative Comparative Analysis
ECs: Ethics Committees
HECs: Hospital Ethics Committees
MECs: Medical Ethics Committees
CNKI: China national knowledge infrastructure
CSSCI: Chinese Social Science Citation Information
CIOMS: International Committee of Medical Scientific Organizations
fsQCA: Fuzzy set qualitative comparative analysis
mvQCA: Multivalue set qualitative comparative analysis
SIDCER: Strategic Action for the Development of Ethics Committee Review Capability
AAHRPP: American Association for Human Research and Protection Project Certification
